# Bone regeneration driven by a nano-hydroxyapatite/chitosan composite bioaerogel for periodontal regeneration

**DOI:** 10.3389/fbioe.2024.1355950

**Published:** 2024-07-30

**Authors:** M. Souto-Lopes, L. Grenho, Y. Manrique, M. M. Dias, J. C. B. Lopes, M. H. Fernandes, F. J. Monteiro, C. L. Salgado

**Affiliations:** ^1^ i3S–Instituto de Investigação e Inovação em Saúde da Universidade do Porto, Porto, Portugal; ^2^ Instituto de Engenharia Biomédica (INEB), Universidade do Porto, Porto, Portugal; ^3^ Faculdade de Engenharia da Universidade do Porto (FEUP), Porto, Portugal; ^4^ Faculdade de Medicina Dentária da Universidade do Porto (FMDUP), Porto, Portugal; ^5^ Laboratório Associado para a Química Verde (LAQV), Rede de Química e Tecnologia (REQUIMTE), Porto, Portugal; ^6^ Laboratory of Separation and Reaction Engineering (LSRE), Laboratory of Catalysis and Materials (LCM), Faculty of Engineering, University of Porto, Porto, Portugal; ^7^ Associate Laboratory in Chemical Engineering (ALiCE), Faculty of Engineering, University of Porto, Porto, Portugal; ^8^ Porto Comprehensive Cancer Center (P.CCC), Porto, Portugal

**Keywords:** bioaerogel, nano-hydroxyapatite/chitosan scaffold, dental follicle mesenchymal cells, osteogenic differentiation, bone regeneration, biomaterials

## Abstract

The most recent progress in reconstructive therapy for the management of periodontitis and peri-implantitis bone defects has relied on the development of highly porous biodegradable bioaerogels for guided bone regeneration. The objective of this work was to evaluate *in vitro* the osteoinduction of periodontal-originating cells (human dental follicle mesenchymal cells, DFMSCs) promoted by a nano-hydroxyapatite/chitosan (nHAp/CS) bioaerogel, which was purified and sterilized by a sustainable technique (supercritical CO_2_). Moreover, the *in vivo* bone regeneration capacity of the nHAp/CS bioaerogel was preliminarily assessed as a proof-of-concept on a rat calvaria bone defect model. The quantification of DNA content of DFMSCs seeded upon nHAp/CS and CS scaffolds (control material) showed a significant increase from the 14th to the 21st day of culture. These results were corroborated through confocal laser scanning microscopy analysis (CLSM). Furthermore, the alkaline phosphatase (ALP) activity increased significantly on the 21st day, similarly for both materials. Moreover, the presence of nHAp promoted a significantly higher expression of osteogenic genes after 21 days when compared to CS scaffolds and control. CLSM images of 21 days of culture also showed an increased deposition of OPN over the nHAp/CS surface. The *in vivo* bone formation was assessed by microCT and histological analysis. The *in vivo* evaluation showed a significant increase in bone volume in the nHAp/CS test group when compared to CS and the empty control, as well as higher new bone formation and calcium deposition within the nHAp/CS structure. Overall, the present study showed that the nHAp/CS bioaerogel could offer a potential solution for periodontal and peri-implant bone regeneration treatments since the *in vitro* results demonstrated that it provided favorable conditions for DFMSC proliferation and osteogenic differentiation, while the *in vivo* outcomes confirmed that it promoted higher bone ingrowth.

## Graphical Abstract

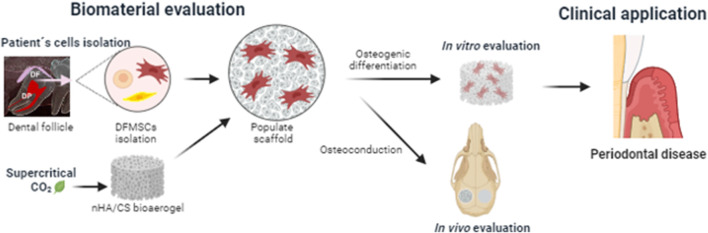

## 1 Introduction

Periodontology is a dental medicine field, which has been developing faster and gaining more worldwide emphasis in recent years, with an increasing impact factor of the related scientific publications (Nazir et al., 2020; Fischer et al., 2021). This is due to the high prevalence of periodontal and peri-implant diseases, which lead to the degradation of the supporting tissues, namely, alveolar bone, and, consequently, to tooth or dental implant loss, respectively (Relvas et al., 2022). Since periodontitis and peri-implantitis severely affect masticatory function, orofacial esthetics, and individual wellbeing (Tonetti et al., 2017), serious efforts have been made in the improvement of new surgical techniques and biomaterials to promote tissue regeneration around teeth and dental implants (Woo et al., 2021). The regeneration of the functional tooth–bone interface requires adequate growth of the acellular cementum, periodontium ligament (PDL), and cryptal bone (Soares et al., 2014). Dental follicle cells (DFCs), which are a type of mesenchymal cells (MSCs) found in periodontal tissues (Liu et al., 2014), are responsible for the building of the interface between bone and dental root during their development. These cells are key players in bone remodeling and periodontium tissue development (Bi et al., 2021). Human dental follicle mesenchymal cells (DFMSCs) can differentiate into osteoblasts and contribute to alveolar tissue formation. Those MSCs have also shown immunomodulatory properties that are instrumental in protecting damaged tissues by releasing anti-inflammatory molecules, reducing fibrosis, and promoting tissue regeneration (da Silva Meirelles et al., 2009). A previous research compared different *in vitro* microenvironments (dynamic vs. static and with vs. without medium supplementation) for DFMSC culture (Salgado et al., 2020), and there is still some uncertainty about the conditions and materials that are more favorable for their proliferation rate and differentiation capacity due to the scarcity of studies.

One of the major applications of bone regeneration biomaterials is in periodontal and peri-implant clinical cases. The commercially available treatments rely on autogenous grafts or on biomaterials that do not always show predictable results in long-term application. Bioaerogels are novel highly porous and biodegradable biomaterials developed using a polymeric matrix from either natural or synthetic origins. Bioaerogels produced with different natural polymers (e.g., polysaccharides and proteins) are safe, economical, and sustainable to be used as biomedical devices (Martins et al., 2015; Goimil et al., 2017; Goimil et al., 2019). Polysaccharides being biocompatible, degradable, renewable, and highly available in nature have garnered great interest in tissue regenerative applications (Verma et al., 2020). Bioaerogels are materials with a large specific surface area and high porosity that possess a highly permeable and appropriate structure to retain large amounts of aqueous fluids (Zheng et al., 2020). Aerogels are solid materials that are obtained after drying, which removes the liquid phase of a gel (being replaced by air) with minimal contraction (Vareda et al., 2018). The outstanding properties of bioaerogels favor cell attachment along with a tunable network of interconnected pores that allows nutrient and oxygen supply to the cells and the disposal of cellular metabolic by-products, making them suitable for application in regenerative medicine (Sehaqui et al., 2011).

The development and production of large-scale biomaterials, in particular, requires decreased use of hazardous compounds and increased use of sustainable sources of materials in order to reduce the environmental impact (Khan and Alamry, 2021). Based on this, chitosan (CS) has garnered great attention due to its availability (from the by-products of seafood industries) (Pitrolino et al., 2022) and pro-regenerative properties such as healing effects, antimicrobial properties, biocompatibility, and biodegradability (Sencadas et al., 2012). Since its degradation produces a harmless amino sugar that could be absorbed by human tissues, CS has a low risk of bioproduct accumulation in important organs such as the liver and kidneys (Levengood and Zhang, 2014; Qasim et al., 2015). Ruphuy et al. (2018) proposed that chitosan-based bioaerogels were produced with freeze drying, followed by exposure to supercritical CO_2_ (scCO_2_), which allowed obtaining a porous structure and extracting 80% of the CS solvent (acetic acid) from the scaffold (Ruphuy, et al., 2018). Unlike other methods for production of chitosan-based scaffolds published in the literature (Tsiourvas et al., 2016; Zhang et al., 2019; Zia et al., 2020; Bozorgi et al., 2022; Cao et al., 2022; Pitrolino et al., 2022; Sadeghianmaryan et al., 2022), the scCO_2_ technique exempted the use of additional solvents and time-consuming washing and drying steps for acid neutralization, resulting in a more sustainable method for scaffold production. Moreover, the scCO_2_ technique allowed us to simultaneously obtain a sterile and ready-to-use final product, as demonstrated in a previous work by a microbiologic assay (Ruphuy et al., 2018). However, CS also showed poor mechanical stability, and to overcome this limitation, a biocomposite was developed by combining this biopolymer with suitable inorganic nanomaterials (nano-hydroxyapatite, nHAp), which provided important characteristics such as higher stiffness and osteoconduction (Ruphuy et al., 2018; Souto-Lopes et al., 2023). One of the major advantages of the presence of nHAp in the composite’s formulation is that it promotes bioactivity, which increases the cell response of forming a direct bond with the bone surrounding tissue (Munir et al., 2022; Zia et al., 2022). In a previous work, the production of the nHAp/CS (70/30%) bioaerogel scaffold was optimized to a simple eco-friendly three-step method (including scCO_2_ solvent extraction and terminal sterilization) (Ruphuy et al., 2018). This 3D scaffold highly resembles the element compositions and structures of native bones (Ruphuy et al., 2018; Souto-Lopes et al., 2023) and simultaneously mimics the structure and chemical properties of bone tissue, which is a composite of ∼70% mineral (mostly hydroxyapatite (HA) nanocrystals) and ∼30% organics (e.g., natural polymers and glycoproteins) (Palmer et al., 2008). In a subsequent study, this developed nHAp/CS bioaerogel showed *in vitro* cytocompatibility, appropriate mechanical behavior for low-load-bearing sites, biodegradability, antimicrobial properties, and *in vivo* biocompatibility in a subcutaneous implant (mouse model) (Souto-Lopes et al., 2023).

To propose the nHAp/CS bioaerogel as an alternative to classic autograft and allograft treatments for bone regeneration of periodontal/peri-implant defects, the present study aimed at exploring, *in vitro*, the scaffold’s osteoinductive and osteoconductive capacity to drive the osteogenic differentiation of DFMSCs, which are periodontal tissue precursor cells with multifunctional properties and excellent potential for regenerative medicine applications. Moreover, as a proof-of-concept, the nHAp/CS scaffold’s capacity to promote bone regeneration *in vivo* was assessed using a simple critical-sized bone defect model before advancing to more complex *in vivo* alveolar bone defect models.

## 2 Materials and methods

### 2.1 Materials

CS (from granules from marine animals’ exoskeleton solution, 90/200/A1, BioLog Heppe, Landsberg, Germany, deacetylation degree 91.9%), nHAp (nanoXIM-HAp102, rod-like nano-particles <50 nm) aqueous paste (Fluidinova S.A., Maia, Portugal), bovine serum albumin (BSA), p-nitrophenol phosphate, p-nitrophenol, sodium hydroxide (NaOH), formaldehyde 4%, Triton X-100, Alizarin Red S, and rabbit anti-human osteopontin were purchased from Merck (Darmstadt, Germany). Dulbecco’s modified Eagle medium (DMEM), fetal bovine serum (FBS), penicillin–streptomycin, and trypsin were purchased from Gibco (Thermo Fisher Scientific, Waltham, MA, United States). The Thermo Scientific™ Pierce™ BCA Protein Assay Kit, Alexa Fluor 488 Phalloidin, Alexa Fluor 488 goat anti-rabbit IgG secondary antibody, and the Quant-iT™ PicoGreen^®^ DNA Assay Kit were purchased from Invitrogen (Thermo Fisher Scientific, Waltham, Massachusetts, United States). PrimePCR™ SYBR^®^ Green Assays (Human GAPDH, SP7, and BMP-2) were purchased from Bio-Rad Laboratories (Algés, Portugal). The NucleoSpin RNA Kit was purchased from Macherey-Nagel (Dueren, Germany). The iScript™ cDNA Synthesis Kit and the iTaqTM Universal SYBR^®^ Green Supermix were purchased from Bio-Rad (Hercules, California, United States). Propidium iodide was purchased from BD Pharmingen ™ (BD Biosciences, Franklin Lakes, New Jersey, United States). Fluoromount VECTASHIELD^®^ Mounting Medium, hematoxylin and eosin, and Masson’s trichrome histological staining kits were purchased from Vector Laboratories (Newark, California, United States).

### 2.2 Preparation of nHAp/CS and CS scaffolds

nHAp/CS scaffolds were prepared using a previously described method (Ruphuy et al., 2018). In brief, a homogenous dispersion was prepared at a 70/30 w/w proportion of, respectively, an nHAp paste (15% w/w, pH 9–10) and a CS solution at 3.0% w/v in acetic acid. The dispersion was poured into 55-mm Petri plates (10 mL in each) and stored at −20°C overnight.

Phase separation was achieved through a standard freeze-drying procedure (VirTis BenchTop 6K, model n°6KBTEL) for 24 h. The residual solvent removal and sterilization were performed by scCO_2_ (an in-house built unit) in continuous batch cycles at 8.0 MPa (80 bar) and 75°C for 2 h. Finally, the individual samples were packed in sterile conditions in Nasco Whirl-Pak^®^ standard bags (2 oz.) and stored at room temperature (RT). Control samples of CS were prepared following the same protocol, except for the addition of the nHAp paste. The scaffolds were cut into smaller samples (cuboid shape of 5 × 5 mm with 4 mm height for *in vitro* experiments; cylindrical shape of 4 mm diameter and 2 mm height for *in vivo* experiments) in sterile conditions.

### 2.3 *In vitro* biological evaluation

#### 2.3.1 Establishment of stem cell cultures from human dental follicle mesenchymal cells

Human dental tissue fragments were isolated from young, healthy patients (approved by the Ethical Committee of the University of Porto—50/CEUP/2018, Porto, Portugal) and fully characterized according to the clinical procedures and laboratory methods described in Supplementary Material of a previous work (Salgado et al., 2020). It was necessary to retrieve follicular sacs from different patients, followed by digestion, adhesion to plastic tissue culture substrates, flow cytometry, and RT-PCR for regular identity assays based on phenotypic and genotypic analyses of the expression of specific MSC markers. After isolation, DFMSCs were selected from a single donor that better fulfilled the predefined criteria such as plastic adherence, phenotypic profile (the presence and absence of specific cell mesenchymal markers), and lineage differentiation (Bieback et al., 2019; Dominici, et al., 2006; Salgado et al., 2020), following the recommendations of the updated guidelines of the International Society for Stem Cell Research (ISSCR) (Daley et al., 2016). Cells were cultured in Dulbecco’s modified Eagle medium supplemented with 10% FBS and 1% penicillin/streptomycin (3 × 10^4^ mol/L and 5 × 10^4^ mol/L) and kept at 37°C in a 5% carbon dioxide (CO_2_) atmosphere (CO_2_ Incubator, Binder, Tuttlingen, Germany). After achieving cell confluence, cells (passage 6) were detached with trypsin solution (0.5%) at 37°C for 5 min and seeded on the scaffolds (0.3 × 10^6^ cells/scaffold). After that, scaffolds were incubated for 7, 14, and 21 days (time points) in similar conditions.

#### 2.3.2 Cellular proliferation assay

DNA content was measured using the Quant-iT™ PicoGreen^®^ DNA Assay according to the manufacturer’s instructions. After each time point, scaffolds were washed with PBS and then incubated with 0.5 mL of ultra-pure water at 37°C and 5% CO_2_ for 1 h. Subsequently, scaffolds were placed in a freezer at −20°C until the end of the experiment and then thawed at RT to lyse all the cell membranes. The supernatant with the lysed cells was collected and incubated with the PicoGreen^®^ solution. Finally, the fluorescence intensity was measured by using a microplate spectrofluorometer (SynergyMx, BioTek, Winooski, Vermont, United States) at 480 and 520 nm excitation and emission wavelengths, respectively. The results are expressed in nanograms of DNA per mL.

#### 2.3.3 Cellular differentiation assay

The alkaline phosphatase (ALP) activity was measured using a quantitative analysis for early osteogenic characterization. The same supernatant with the lysed cells obtained as described above (Section 2.3.2) was used for assessment of the enzyme activity and total protein content. The ALP enzyme activity was assessed by monitoring substrate hydrolysis using p-nitrophenol phosphate in an alkaline buffer solution (pH = 10). After 1 h of incubation at 37°C, the reaction was terminated by adding NaOH (0.02 M), and the p-nitrophenol was quantified by absorbance at 405 nm using a plate reader (Synergy MX, BioTek, Winooski, Vermont, United States). Finally, the ALP results were expressed in nanomoles (nmol) of p-nitrophenol produced per minute (min). The ALP activity results were normalized to the total DNA content (cell density) and expressed in nanomoles of p-nitrophenol produced per minute per µg of DNA.

Total protein content was measured by Lowry’s method (Thermo Scientific™ Pierce™ BCA Protein Assay Kit) with bovine serum albumin used as the standard. Results were expressed in milligrams of protein concentration per mL.

The expression of relevant osteogenic genes was monitored throughout the 21-day culture and analyzed by real-time quantitative polymerase chain reaction (RT-qPCR) at the time points 14 and 21 days. In brief, total RNA was extracted using a NucleoSpin RNA Kit and reverse-transcribed into complementary DNA (cDNA) using the iScriptTM cDNA Synthesis Kit, as per the manufacturer’s instructions. The expression of the target genes was quantitatively determined on RT-PCR equipment (CFX384, Bio-Rad, Hercules, CA, United States) using the iTaq™ universal SYBR^®^ Green Supermix. All genes were normalized to the reference gene glyceraldehyde 3-phosphate dehydrogenase (*GAPDH*) and are described in Table 1. Relative quantification of gene amplification by qPCR was performed using the cycle threshold (Ct) values, and relative expression levels were calculated using the 2^−ΔΔCT^ method. DFMSCs cultured at time 0 were used as a normalizer for the osteogenic gene expression (value 1). A 2D control (tissue culture plate) was used for 14 days and 21 days. For each qPCR, samples were analyzed in duplicate, and three independent experiments were performed.

**TABLE 1:** Gene name and respective primers for RT-qPCR.

Gene	Primer assay ID (Bio-Rad)	
***GAPDH***	qHsaCED0038674	
***SP7***	qHsaCED0003759	
***BMP-2***	qHsaCID0015400	

Primer sequence (forward)		Primer sequence (reverse)
***OPN***	5’—ACT​CGA​ACG​ACT​CTG​ATG​ATG​T—3′	5’—GTC​AGG​TCT​GCG​AAA​CTT​CTT​A—3′
***Col-1***	5’—TCC​GGC​TCC​TGC​TCC​TCT​TA—3′	5’—ACC​AGC​AGG​ACC​AGC​ATC​TC—3′

#### 2.3.4 Confocal laser scanning microscopy

Samples from each time point were fixed with 4% paraformaldehyde and incubated for 30 min at RT. Then, the materials were incubated with 0.1% Triton X-100 solution and then 1% BSA solution to enhance sensitivity by reducing background interference. The cell cytoplasm (actin fibers) was stained with Alexa Fluor-conjugated Phalloidin 488 nm (dilution of 1:150) for 1 h, and nuclei were stained with propidium iodide (1 mg/mL) for 10 min at RT and under dark conditions. For human osteopontin immunostaining, an identical protocol was employed for the cell membrane’s permeabilization and to block nonspecific binding, as described above. Samples were then incubated with rabbit anti-human osteopontin (AB 1870, 1:500) overnight at 4°C. This procedure was followed by 1 h incubation with Alexa Fluor 488 goat anti-rabbit IgG secondary antibody (1:1000). All samples were covered by Fluoromount. Images were acquired with excitation lasers of 405 (CS autofluorescence), 488 nm, and 594 nm and evaluated by confocal laser scanning microscopy (CLSM, Leica SP2 AOBS SE Camera, Leica, Wetzlar, Germany).

### 2.4 *In vivo* evaluation

#### 2.4.1 Animal model

**FIGURE 1:**
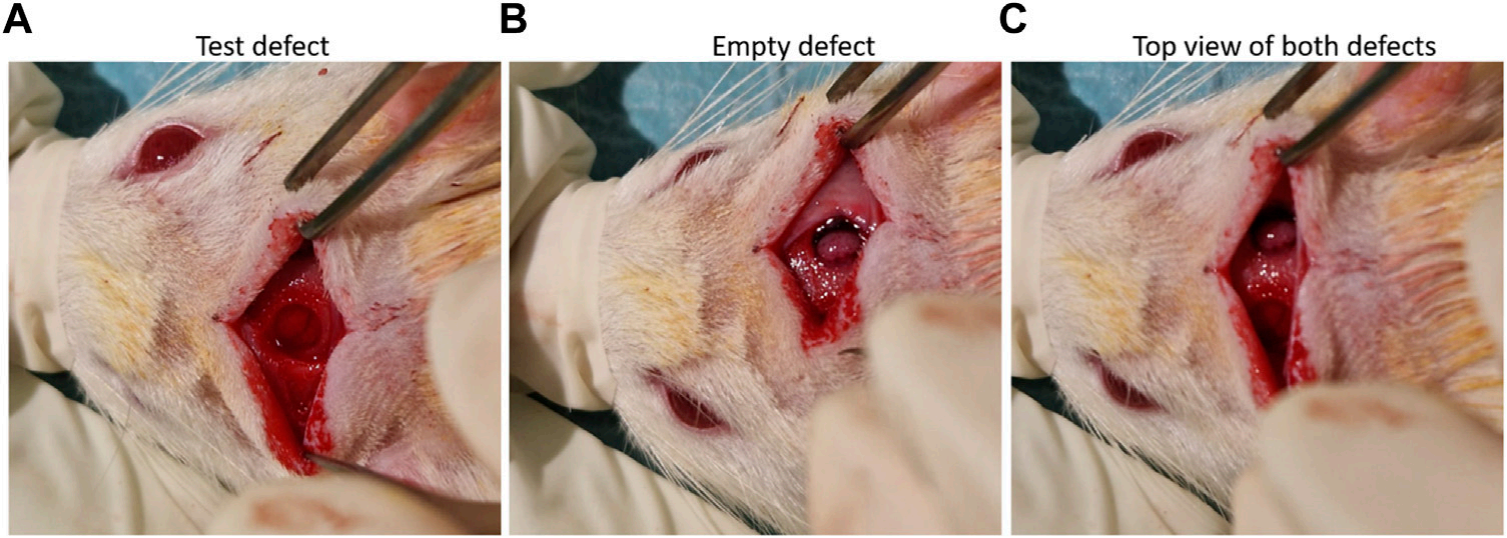
**(A)** nHA/CS scaffold implant, **(B)** view of the empty defect, and **(C)** both defects created in the rat calvaria.

nHAp/CS and CS scaffolds were implanted into the calvarial bone of 10-week-old male Wistar rats (12 animals; i3S animal house, Portugal). Based on the results in other published reports, G*Power software was used to estimate the minimum number of animals needed for the study (n = 6 for each tested group (nHA/CS and CS) with an effect size of 2.12 for paired samples). All animal experiments were approved by the i3S animal ethics committee (EC) and by DGAV (Portugal). All tests followed EC guidelines for animal welfare. Researchers involved in animal handling were FELASA-accredited and DGAV-certified. Animals were anesthetized with 3%–5% isoflurane for induction and 1%–2% for surgical procedures that were performed under standard aseptic conditions. A midline incision was performed through the parietal midline skin, and two 4 mm (diameter) bone defects were created, one on the right side (control—empty defect) and another one on the left side (scaffolds) (Figure 1). An *in vivo* microCT scan was performed after 3 days (control) and 1, 2, and 3 months to follow the bone volumetric changes.

In brief, the animals were individually placed in an induction chamber, and anesthesia was induced at 3%–5% and maintained with 1%–2% isoflurane during *in vivo* assessment in the microCT device (Bruker SkyScan 1276, Bruker Corporation, Billerica, Massachusetts, United States). Data were collected from a region of interest (ROI) shaped like a cylinder with a diameter of 4 mm and thickness of 1.5 mm, based on the size of the created defect area. 3D images of the defects were obtained, and the quantitative parameters calculated were bone volume (BV—mm^3^), bone volume *versus* tissue volume (BV/TV—%), and bone surface (BS—mm^2^).

After recovery, the rats were caged in pairs and allowed to move without restriction. They were fed with commercial rat chow and water for 3 months *ad libitum*. After the required period, the animals were euthanized with carbon dioxide asphyxiation.

#### 2.4.2 Histology analysis

All samples were explanted and fixed in 10% neutral buffered formalin for 3 days and then processed for histology (Paraffin Tissue Processor Microm STP 120-1, Thermo Fisher Scientific, Waltham, MA, United States). Fixed samples were decalcified according to the manufacturer’s instructions (Shandon TBD-1™ Decalcifier, Thermo Fisher Scientific, Waltham, MA, United States), embedded in paraffin according to the manufacturer’s instructions (Modular Embedding System Microm, Thermo Fisher Scientific, Waltham, MA, United States), and sectioned transversally into 5 mm-thick slices (Paraffin Microtome Microm HM335E, Thermo Fisher Scientific, Waltham, MA, United States) and stained with hematoxylin and eosin, while the longitudinal sections were stained with Masson’s trichrome and Alizarin Red S (calcium deposition) for light microscopy examination.

### 2.5 Statistical analysis

Data were presented as the mean and standard deviation and analyzed using the two-way ANOVA test for *in vitro* experiments and one-way ANOVA for *in vivo* experiments (GraphPad Software, Insight Venture Partners, New York City, NY, United States). Differences between groups and time points were considered statistically significant when *p* < 0.05.

## 3 Results

### 3.1 *In vitro* evaluation

**FIGURE 2:**
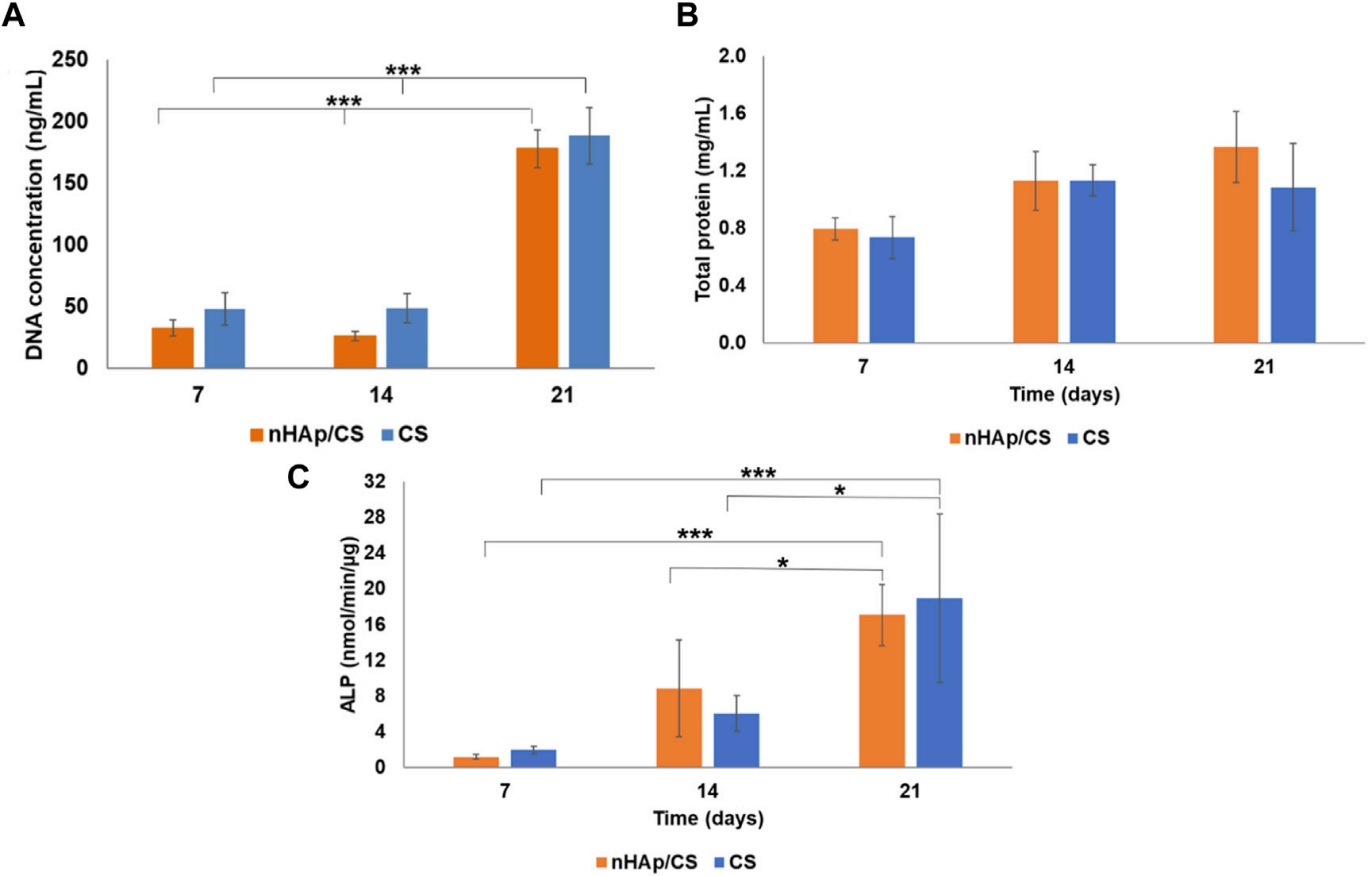
**(A)** Total DNA content, **(B)** total protein, and **(C)** ALP activity of DFMSCs cultured within nHAp/CS biocomposite and CS scaffolds for 7, 14, and 21 days. Statistical differences between samples from different time points. *p < 0.05 and ***p < 0.001.

DFMSCs were seeded into nHAp/CS and CS scaffolds and evaluated after 7, 14, and 21 days (Figure 2A). Few cells were observed in both materials after 7 and 14 days. However, on day 21, both scaffolds showed a statistically higher cell number (4-fold cellular increase). Protein content increased from days 7 to 14, being approximately stable afterward (Figure 2B). ALP activity increased throughout the culture time, and on day 21, values were significantly higher than those measured on days 7 and 14 (Figure 2C). The three parameters were similar in nHAp/CS and CS scaffolds throughout the evaluation time.

**FIGURE 3:**
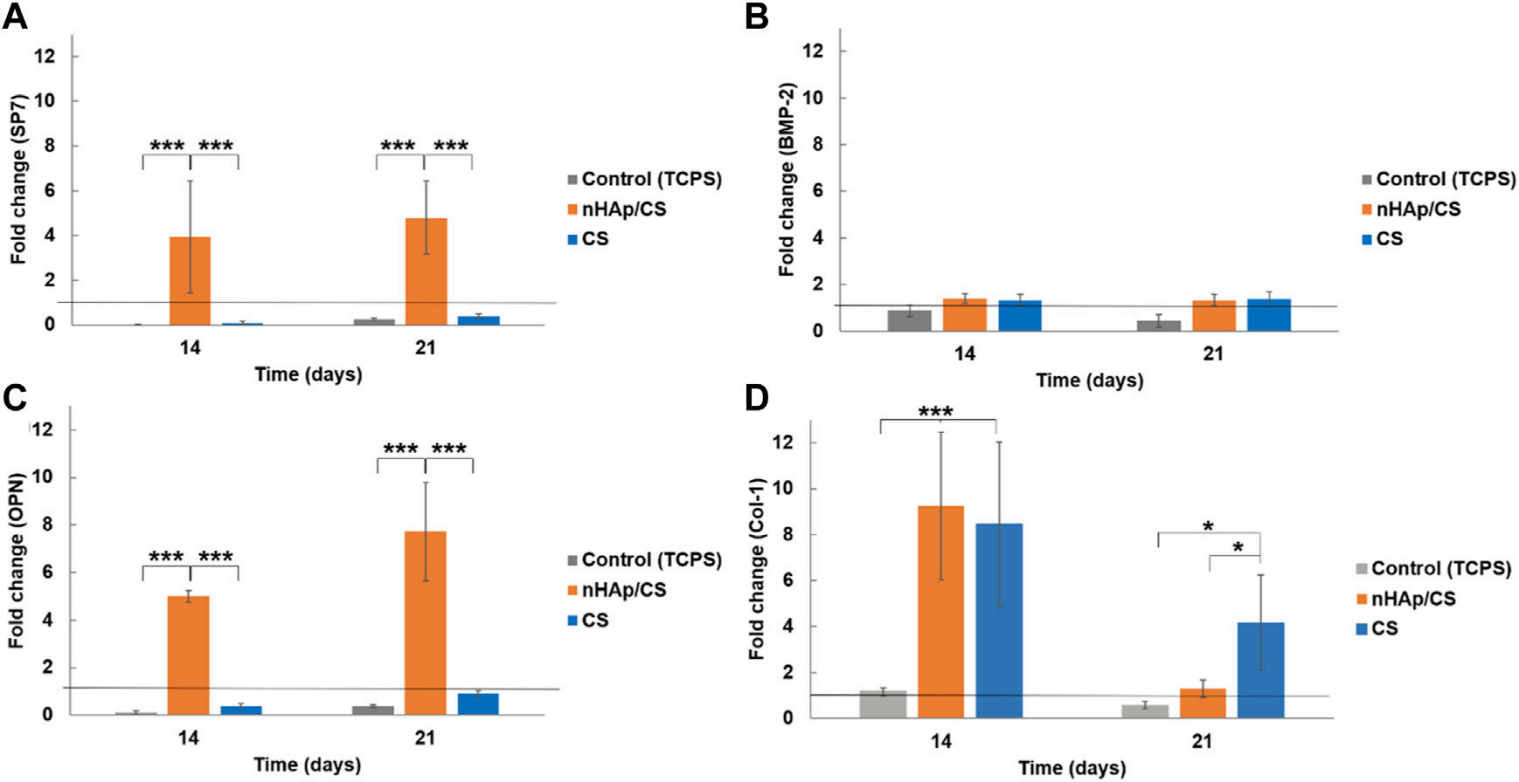
Quantitative real-time polymerase chain reaction (qPCR) for the osteogenic genes (*Osterix* gene, SP7 **(A)**; bone morphogenetic protein 2, BMP-2 **(B)**; osteopontin, OPN **(C);** and collagen 1, Col-1 **(D)** for DFMSCs cultured within the nHAp/CS or CS scaffolds for 14 and 21 days. Quantitative data were calculated by the ΔΔCt method using *GAPDH* gene expression as an endogenous reference. Sample results were normalized to the 2D (standard culture plate, black horizontal line) cultured cells (average results). These are represented as fold changes. Statistical analysis: *p < 0.05 and ***p < 0.001.

The results obtained for the expression of osteopontin (OPN), Osterix (SP7), bone morphogenetic protein 2 (BMP-2), and collagen 1 (Col-1) on days 14 and 21 of culture are shown in Figure 3. Cultures performed within the nHAp/CS scaffold presented a significantly higher expression of SP7 (coding for Osterix) (Figure 3A) and OPN (Figure 3C) when compared to the gene expression of cells in the CS scaffold and the control. The gene expression increased approximately 4-fold for SP7 and 5-fold for OPN on day 14. On day 21, the high expression of these genes in nHAp/CS was maintained, i.e., 5-fold for SP7 and 7.5-fold for OPN. The expression of BMP-2 was similar to the undifferentiated DFMSCs’ expression for all materials and control at both time points. Col-1 expression was similar for both biomaterials after 14 days and decreased on day 21, but both scaffolds were statistically different from the control throughout the time (Figure 3B).

**FIGURE 4:**
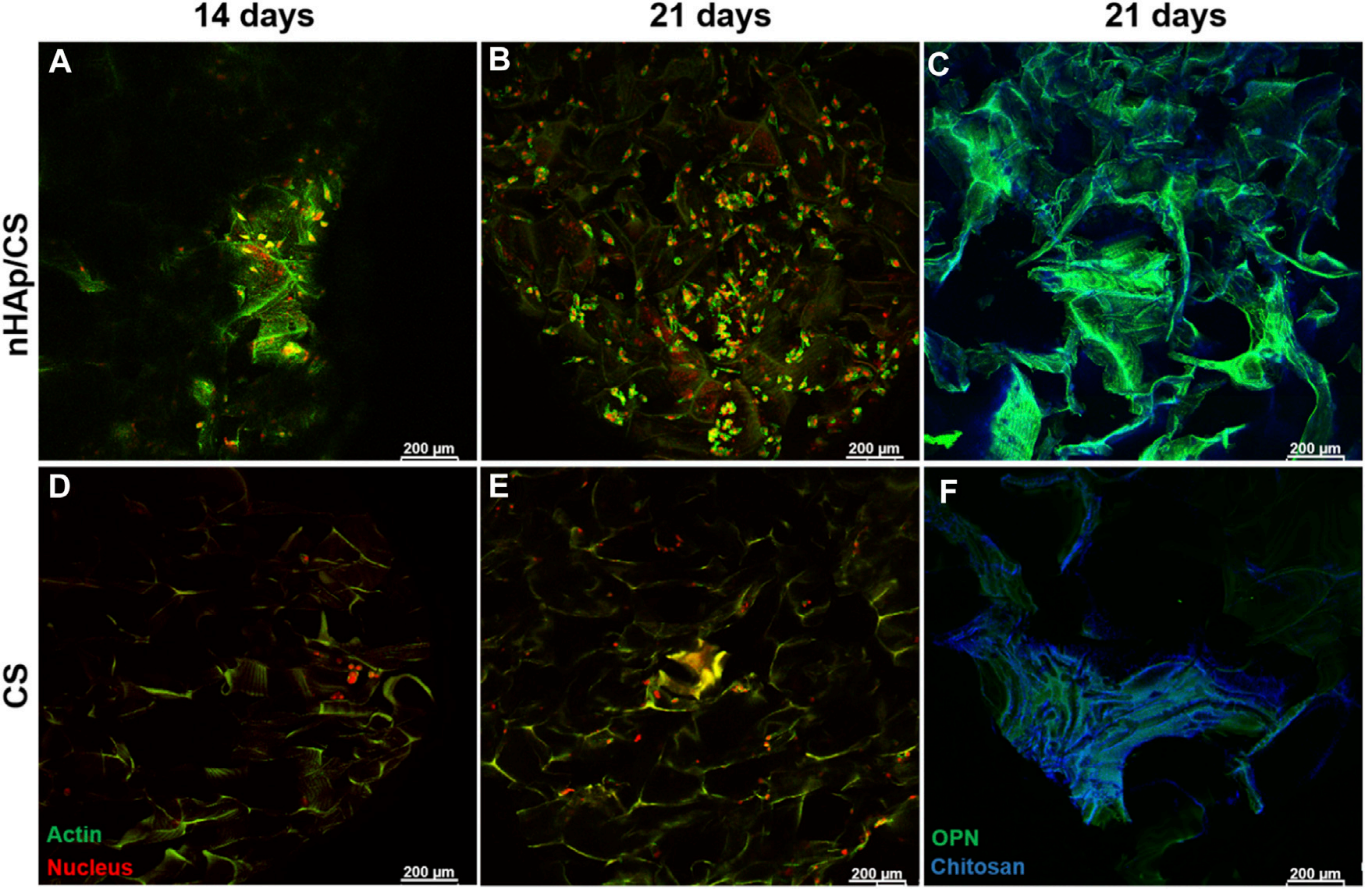
CLSM images showing the DFMSC morphology (staining for actin cytoskeleton and nucleus **(A, B, D, E)**) and human osteogenic ECM (staining for OPN; **(C, F)**) after 14 and 21 days of culture within the nHAp/CS or CS scaffold. Actin cytoskeleton, OPN (green), and nucleus (red); blue staining refers to chitosan autofluorescence. Scale bar: 200 μm.

The cellular morphology and the human proteins secreted by DFMSCs were evaluated by the immunostaining of cytoplasmic actin and OPN after 14 and 21 days of culture within the scaffolds. The results are shown in Figure 4. Cultures stained for the actin cytoskeleton showed low cell colonization within both scaffolds on day 14, appearing mostly as cellular aggregates (Figures 4A, D). Images of the 21st day of culture showed a higher number of cells in the nHAp/CS scaffolds (Figure 4B) when compared to the CS scaffold (Figure 4E). Furthermore, cells exhibited higher cell volume, a well-identified nucleus, and cytoplasm, and cell colonization was visible throughout the composite scaffold (Figure 4B), when compared to CS samples (Figure 4E).

The osteopontin deposition on the 21st day of culture showed a larger amount of this protein over the nHAp/CS scaffold surface than that over CS scaffolds (Figures 4C, F). These results corroborate the OPN gene expression shown in Figure 3.

### 3.2 *In vivo* evaluation

#### 3.2.1 microCT quantitative analysis

**FIGURE 5:**
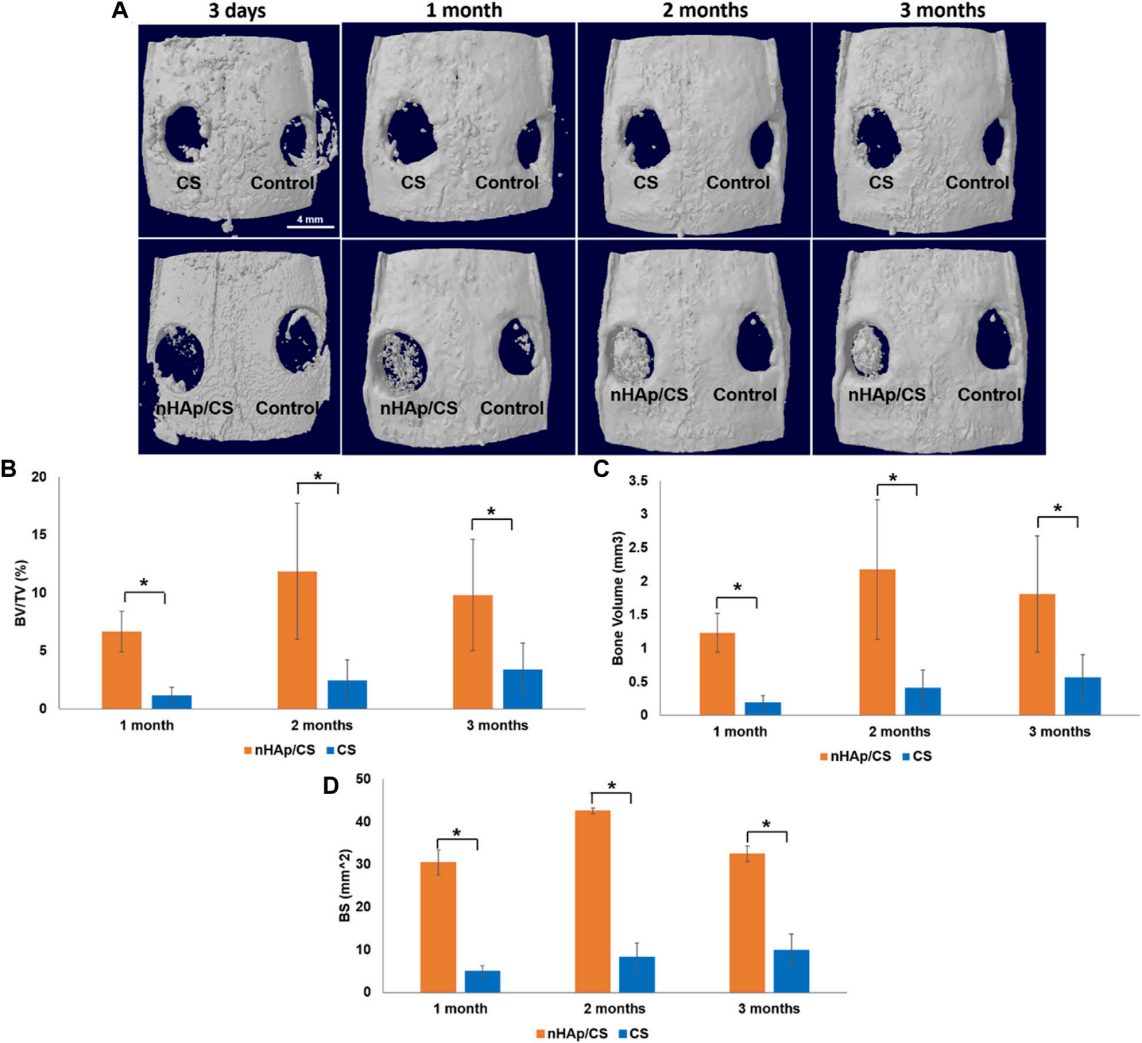
**(A)** Top defect views of 3D reconstructed microCT image analysis showing the degree of bone repair in empty defects (control), nHAp/CS and CS scaffolds implanted into the rat calvaria after 3 days and 1, 2, and 3 month post-surgery (scale bar 4 mm). **(B)** Quantitative microCT data analysis of the bone level/tissue level ratio (%). **(C)** Quantitative microCT data analysis of bone volume (mm^3^). **(D)** Quantitative microCT data analysis of the bone surface (mm^2^). Statistical analysis: *p < 0.05.

nHAp/CS and CS scaffolds were implanted in a calvarial bone critical defect model, and *in vivo* microCT imaging was performed after 3 days and 1, 2, and 3 months (Figure 5A). Two animals died after surgery, and a total of ten animals underwent microCT scans. Quantitative analysis was further displayed, and the BV was significantly higher in the nHAp/CS group than in the CS group: 1.23 mm^3^ (BV/TV: 6.69%; BS: 40.3 mm^2^) *versus* 0.19 mm^3^ (BV/TV: 1.15%; BS: 5.0 mm^2^), 2.18 mm^3^ (BV/TV: 11.88%: BS: 42.6 mm^2^) *versus* 0.41 mm^3^ (BV/TV: 2.46%; BS: 8.3 mm^2^), and 1.81 mm^3^ (BV/TV: 9.84%; BS: 32.5 mm^2^) *versus* 0.57 mm^3^ (BV/TV: 3.41%; BS: 9.7 mm^2^), respectively, after 1, 2, and 3 months (Figures 5B–D).

#### 3.2.2 Histological analysis

**FIGURE 6:**
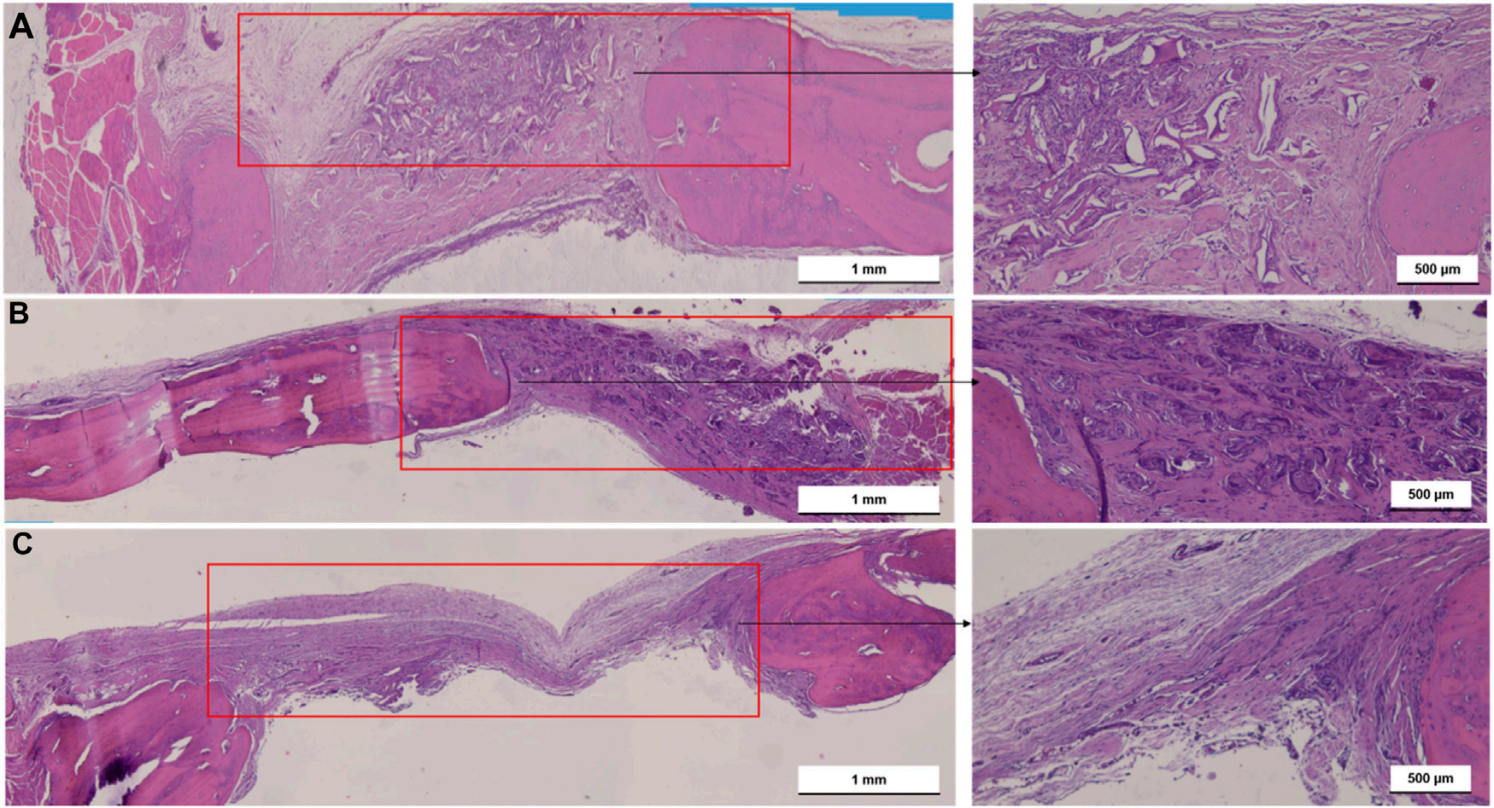
Light microscopy images of nHAp/CS **(A)**, CS **(B)** scaffolds implanted for 3 months and empty bone defects as control **(C)**. Transversal slides were stained using H&E. Scale: 500 μm and 1 mm. Red square, bone defect.

Figure 6 shows one set of representative ground sections per group of the calvarial defect, corresponding to the median transversal slices (stained by H&E) of nHAp/CS (a), CS (b), and control (empty bone defect (c)), in terms of bone regeneration, that were in accordance with the microCT analysis. Higher bone formation was also observed inside the nHAp/CS scaffolds and in the surrounding tissue (defect borders) (Figure 6A). On the contrary, bone formation was observed only around the CS scaffolds after 3 months (Figure 6B). The empty bone defect in the control group was mainly filled by connective tissue (Figure 6C). The qualitative imaging analysis revealed that in the central compartment of the defect, the bone formation area was significantly higher in the nHAp/CS group than in the CS group and control (empty defect) (Figure 6).

**FIGURE 7:**
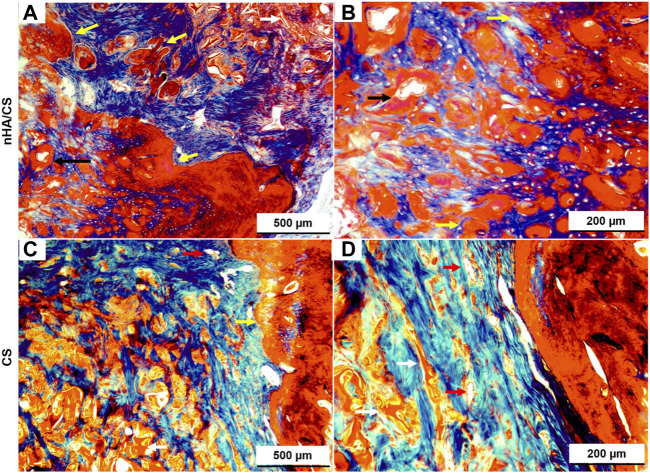
Light microscopy images of nHAp/CS **(A, B)** and CS **(C, D)** scaffolds implanted for 3 months. Longitudinal slides were stained using Masson’s trichome. Scale: 500 and 200 µm. White arrows, scaffolds; black arrows, blood vessels; and yellow arrow, new bone tissue.

High bone formation inside the nHAp/CS was confirmed by the longitudinal section (stained by Masson’s trichome, Figures 7A, B). The fibers of the original scaffold structure (dark pink) were either surrounded by the new bone or soft tissue. This new bone formed trabecular ridges with random orientation, and it was enclosed by thin layers of parallel-trabecular bone. On the contrary, fibrous tissue with random collagen orientation could be observed inside the CS scaffold (Figures 7C, D, dark blue color).

**FIGURE 8:**
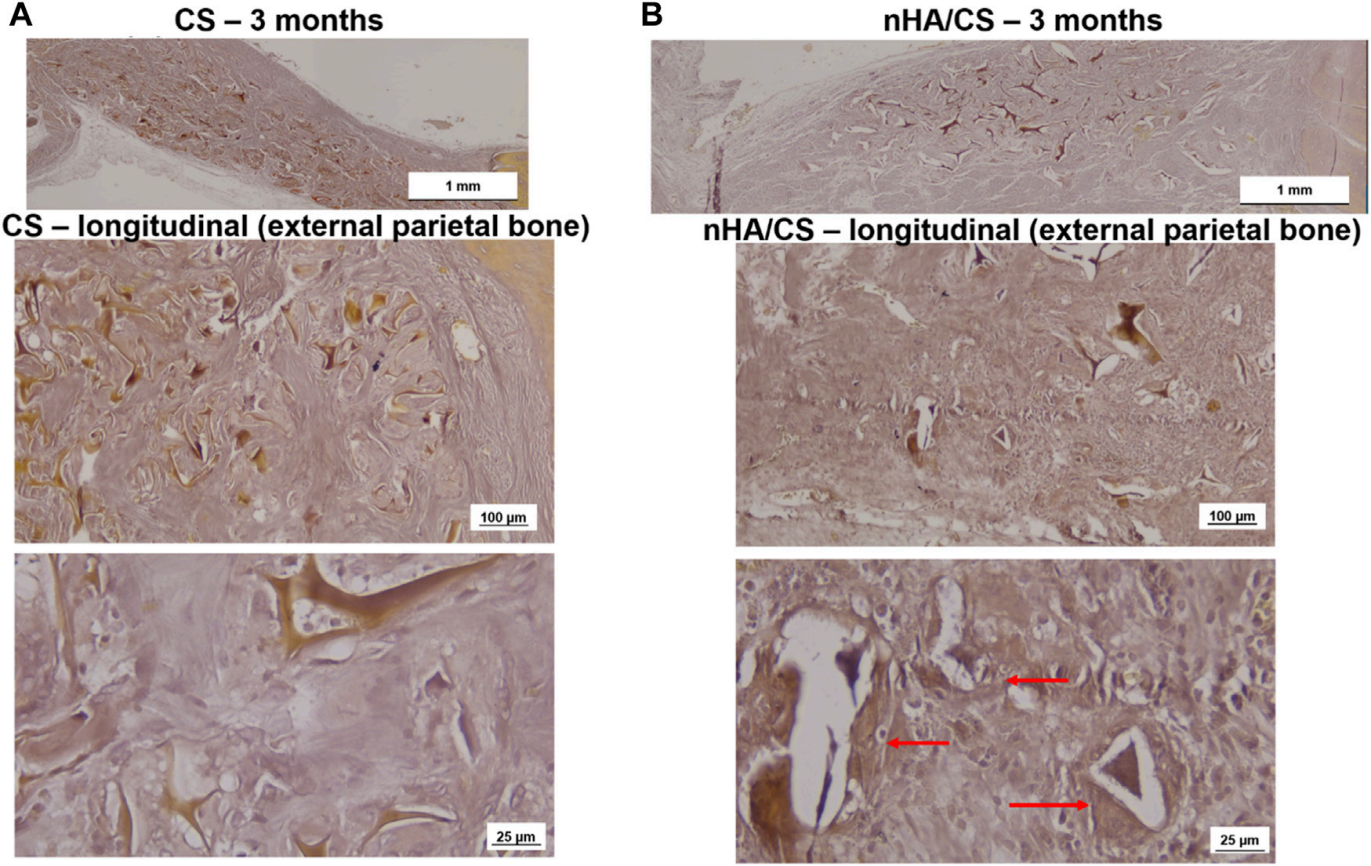
Light microscopy images of CS **(A)** and nHAp/CS **(B)** scaffolds implanted for 3 months. Longitudinal slides (external parietal bone) were stained using Alizarin Red S. Scale: 1 mm, 100 µm, and 25 µm. Red arrows, calcium deposition circumventing the nHAp/CS biomaterial.

The longitudinal slides of the external parietal bone in Figure 8 (stained by Alizarin Red S) show that the deposition of calcium was only visible around the nHAp/CS biomaterial (Figure 8B) since no calcium deposition was observed in the CS scaffolds (Figure 8A).

## 4 Discussion

The present work compared the osteogenic potential of nHAp/CS with that of CS bioaerogel scaffolds, as possible graft biomaterials to fill bone defects in low-load-bearing sites such as in periodontal and peri-implant regenerative treatments. The scaffolds’ composition and surface properties are key factors in achieving bone tissue regeneration with adequate MSC osteogenic differentiation (Granz and Gorji, 2020). However, MSC isolation for *in vitro* testing usually requires an invasive surgical procedure. Furthermore, MSCs experience a progressive decline in regeneration and differentiation capacities with aging (Oh et al., 2014). To overcome these limitations, human dental MSCs, in particular follicle-derived MSCs, have received increased interest in the field of regenerative medicine since they can be isolated from unerupted and impacted teeth, which are usually discarded as dental medical waste, with no extra surgery being required, providing economic value for waste-derived tissue (Rezai-Rad et al., 2015). Furthermore, these neural crest-originated cells have other advantages such as high viability and proliferation rate (Patil et al., 2014), active self-renewal capability, immunomodulatory properties, feasible cryopreservation, and absence of ethically related issues (Mori et al., 2012; Bi et al., 2021). DFMSCs also have a multipotent differentiation capacity with high pluripotency and plasticity since they can differentiate into osteoblasts, chondrocytes, adipocytes, neuronal cells, and different dental cells such as periodontal ligament (PDL)-type lineages (Zhang et al., 2019). Therefore, DFMSCs have strong osteogenic capability to differentiate into the osteoblastic lineage (Morsczeck, 2022). Graziano et al. (2008) confirmed that dental MSCs are a promising source for bone tissue regeneration due to their high capacity to adhere to biomaterial surfaces.

DNA quantitative analysis is a simple and accurate *in vitro* test that quantifies the number of cells in a sample and gives a glimpse of the cellular proliferation rate. The higher the DNA concentration, the higher the cell number and, subsequently, the higher the proliferation rate. The DFMSCs cultured into nHAp/CS or CS scaffolds displayed similar DNA concentration, which significantly increased during long culture times, i.e., on day 21, and appeared to be a consequence of the cell aggregation observed at the lower time points (on days 7 and 14), as confirmed by CLSM images. The initial lower proliferation rate (on days 7 and 14) could be explained by the CS surface characteristics. The positive surface charge arising from protonated amino groups in CS is known to predict cell adhesion. The lack of negatively charged groups on the CS surface for interactions with the positively charged amino groups of proteins could be the reason for the poor cell adhesion, on chitosan membranes, as reported previously by Reis and co-workers (López-Pérez et al., 2007; Silva et al., 2008) and other authors (Chung et al., 2002; Cuy et al., 2003; Li, et al., 2006).

The presence of bioceramic nanoparticles in bone scaffolds has been shown to provide excellent bioactivity, which promotes bone tissue development (Lemos et al., 2022). During regeneration of mineralized tissues such as bone, the mineralization processes are triggered by the presence of other negatively charged groups, the phosphate groups. The importance of these groups has been recognized by the biomaterial research community for a long time, and calcium phosphate materials are commonly used in bone regenerative medicine. This was our hypothesis, although the CS matrix covered the HAp nanoparticles and its low degradation rate would allow the release of the ceramic only after 14 days since the cell behavior changed after 14 days. This new hypothesis is in accordance with the results of the ALP enzyme activity that increased only after the 14th day. ALP is highly expressed in the cells of mineralized tissues, and, *in vitro*, its activity is higher in the initial phases of the osteoblastic differentiation process (Vimalraj, 2020). In the present work, the DFMSCs’ ALP activity was similar in both materials, as was the expression of the BMP-2 gene. The activation of the BMP-2 signaling pathway shall control the ALP expression and lead to osteoblast differentiation and higher ALP activity (Vimalraj, 2020). The upregulation of BMP-2 could be induced by N-acetylglucosamine (the degradation product of CS), which promotes osteoblast activity and an increase in the expression of BMPs (Nagel et al., 2013). The total protein content was quantified to provide information on the ECM production, which also showed an increased tendency until day 21 (not statistically different) with the nHA/CS scaffold, demonstrating again DFMSC differentiation. Instead of the total protein content, another parameter that could influence cell differentiation is the increase in one of the bone ECM’s components, osteopontin, after 14 and 21 days. At those time-points, fluorescence microscopy observations showed DFMSC images showing spindle-like morphologies within the porous structure of the nHAp/CS scaffold, which shows that the surface’s chemical composition, topography, and energy are more favorable for osteoconduction compared to the plain CS scaffold (Figure 4). Pitrolino et al. (2022) also achieved a higher number of cells attached to the nHAp/CS scaffold surface, while at the CS-only scaffold, the cells exhibited clusters with a more rounded morphology (Pitrolino, et al., 2022). Bozorgi et al. (2022) reported that a favorable Saos-2 cell morphology (with extended filopodia) was observed after seeding those pre-osteoblast cells on a nHAp/CS/Gel scaffold for 3 days of analysis (Bozorgi, et al., 2022). Zhang et al. (2019) also tested hDPMSCs seeded on their chitosan/poly (γ-glutamic acid)/hydroxyapatite (CPH) hydrogel (or without HAp) scaffolds. They found that besides an increase in the metabolic activity from 24 to 72 h, the cells showed a polygonal morphology and spread with multiple filopodia contacts during microscopy observation. The presence of bioactive ceramics serves as topographical cues, promoting cellular interaction with the biomimetic surface of the scaffold and allowing focal cell adhesions, which not only enhance adhesion itself but also the formation of filopodia and cellular spreading and, consequently, osteointegration (Munir et al., 2022). The results from previous work with the nHAp/CS scaffold showed that apatite crystals start to precipitate *in vitro* upon the surface after 7 days of incubation in SBF, evolving into needle-like crystals after 21 days (Souto-Lopes et al., 2023), which also explains the delay in DFMSC attachment and proliferation.

Our qPCR results are expressed as a function of the fold change (FC), which relates gene expression obtained for each scaffold and by the control (cells cultured in 2D conditions, i.e., the standard culture plate). Thus, an FC greater than 1 implies that gene expression is greater than that in the control. Therefore, qPCR results also clearly showed that the nHAp/CS biocomposite triggered higher cellular differentiation, as evidenced by the observed osteoblastic gene expression profile and OPN immunostaining of the colonized scaffolds. Thus, DFMSCs cultured in the nHAp/CS composite displayed significantly higher expression of *SP7* and *OPN* genes. The *SP7* gene is associated with the osteoblastic phenotype, being the gene coding for the late osteogenic transcription factor Osterix. This factor regulates and induces the expression of a set of mature osteoblastic genes coding for the synthesis of late ECM proteins involved in terminal osteoblastic differentiation, including OPN (Liu et al., 2020). OPN is a major non-collagenous ECM structural protein, being part of the organic component of bone. Its expression mainly occurs in osteoblastic-lineage cells, and it is expected to be associated with the induction of osteogenic differentiation. A study showed that the presence of OPN in the material played a vital role in the recruitment of MSCs during tissue regeneration (Wang et al., 2017), as well as promoting cellular differentiation into the early pre-osteoblast phenotype (Costa et al., 2023). Furthermore, CLSM images of the colonized nHAp/CS and CS samples clearly evidenced a significantly higher OPN deposition in the composite bioaerogel. It appears that OPN binds tightly to hydroxyapatite (HAp) and seems to form an integrated part of the mineralized matrix. In the bone repair process, hydroxyapatite plays a key role in the proliferation of osteoblasts (Zastulka et al., 2023). In addition, CS contributes to osteoblast differentiation and bone healing (Tian et al., 2022). Control cells, corresponding to DFMSCs at passage 6 seeded in a 24-well tissue culture plate, were harvested on day 0 for RNA isolation and purification assays. In this passage, Costa et al. (2023) showed that the MSCs maintain the mesenchymal phenotype, and the 2^−ΔΔCT^ method was used as a normalizer for osteogenic gene expression (value 1 in the graphs, Figure 3). Since DFMSCs in osteoinductive medium/biomaterials were able to start differentiation after 14 and 21 days (shown by the increased expression of those osteogenic genes), they showed the osteoblast phenotype. A 2D control was added to the qPCR analysis for 14 and 21 days to provide additional information about the difference in gene expressions of DFMSCs cultured at 2D or 3D, as also shown in a previous work (Figure 3 of the supplementary results of Salgado et al. (2020)). The control (2D) results were similar to or below 1 in Figures 3B, D indicating that the cells were still expressing the mesenchymal phenotype and did not start to differentiate into the osteoblast phenotype.

The *in vivo* experiments of this research were performed in a well-established small animal model (the calvaria of Wistar rats) as a proof-of-concept of the osteogenic potential of the nHAp/CS bioaerogel before proceeding into more complex bone defects such as those found in the periodontal context. Creating that kind of critical-sized defects in small animal models such as the rat would be visually challenging and could result in iatrogenic lesions such as tooth necrosis, compromising the masticatory function and causing unnecessary morbidity to the animals (Giannobile and Nevins, 2011; Han, et al., 2013). Therefore, it was possible to create two critical-size defects in each animal, allowing for a decrease in the number of animals used in a site (calvaria) subjected to relatively low loads. When considering the histologic section and microCT, some of the newly formed bone was located inside and outside the defect margin and within the space created by the scaffold into the defect area. Similar results were shown by Strauss and collaborators with collagen membrane implants (Strauss et al., 2021). The bone regeneration was significantly advanced in the nHAp/CS group compared to the CS and control groups (empty defect). Interestingly enough, CS had no impact on bone formation but served just as a template for cell proliferation (Pattnaik et al., 2011), but nHAp adsorbs proteins and other biomolecules, releasing calcium and phosphate ions and acting as an osteoconductive carrier (Chesnutt et al., 2009). This osteoconductive activity is supported by the regeneration pattern displayed in the microCT images, suggesting that the nHAp/CS scaffold is not a passive porous material, as was observed with the CS scaffold. Another study using polymeric gel showed in the histological examination that there was a tendency for new bone to be formed near the cranial dura matter side of the bone defect, above the site where new blood vessels were formed (Kurobane et al., 2019). Similar bone tissue growth was observed with nHAp/CS implants by microCT images (inner bone formation, Figure 5A) and histology, where newly formed vessels surrounded by mineralized tissue (early angiogenesis, Figure 6A; Figure 7B) being promoted by the biocomposite would also contribute to bone regeneration. These observations, together with the histological analysis (Masson trichrome staining), suggested that the bone regeneration was not restricted to the area of the scaffold modified with nHAp, but it was also observed in the borders of the bone defect, and in the absence of the nanoparticles, the biomaterial is fully filled with scar tissue (CS scaffold and empty defect control). Taken together, these findings show that nHAp presence in the scaffold composition plays an important role in the neo-formation of a mineralized ECM and induced bone formation in a rat calvarial critical defect model. Cao et al*.* (2022) also observed higher bone growth for their CS/nHAp scaffold after 3 months (Cao et al., 2022). Chatzipetros et al. (2021) observed a significantly higher fraction of bone regeneration (FBR) from the second to the eighth week for the HAp/CS 75/25 w/w scaffolds (19.96% vs. 42.13%) than for empty controls (14.88% vs. 15.98%), after histomorphometry evaluation of the bilateral 5-mm defects on rat calvaria (Chatzipetros et al., 2021). The chitosan/poly (γ-glutamic acid) (CP) scaffolds, reinforced or not with hydroxyapatite (CPH) and enriched with platelet-rich fibrin (CPH-PRF), developed by Zhang et al. (2019), were first tested in 5 mm calvaria defects in rats, and by the eighth week, all groups showed a large amount of newly formed bone, though the CPH−PRF group exhibited a significantly higher bone repair effect (mean integrated density of ∼70% of control, evaluated by microCT) (Zhang, et al., 2019).

It has been reported that the porous structure of the CS scaffold, particularly after lyophilization, provided lower mechanical stability, having a negative impact on bone regeneration (Mohammadi et al., 2016). Therefore, the combination of CS with nHAp resulted in a reinforced structure, increasing its potential to successfully promote bone cells and MSC proliferation and differentiation, allowing bone tissue growth within the bone defect site (Pighinelli and Kucharska, 2013). However, in a previous study, we observed that both the nHAp/CS and CS scaffolds implanted in subcutaneous pockets in mice showed stable structures after 5 weeks post-implantation, probably due to the high deacetylation degree of the CS used to produce the bioaerogels (Souto-Lopes et al., 2023). As can be observed in the histologic images (Figures 6–8), even at 3 months post-surgery, the structures of both scaffold types are still visible.

Despite the advantages of the nHAp/CS scaffold demonstrated in the present work, there are still more specific experiments that would be necessary in order to achieve alveolar bone regeneration, such as additional studies on the bioaerogel antimicrobial effect against anaerobic oral bacteria species and periodontopathogens in particular. It would also be important to evaluate, *in vitro,* the nHAp/CS scaffold angiogenic potential and to create defects in the alveolar bone in *in vivo* experiments with larger animal models to study more reliably the clinical application of the nHAp/CS scaffolds in order to achieve a bone graft biomaterial suitable for clinical use in the field of periodontology.

## 5 Conclusion

This work showed encouraging *in vitro* and *in vivo* results obtained with the nHAp/CS scaffolds produced with a low environmental impact and an eco-friendly process. The study supported that the nHAp/CS bioarogel increased the *in vitro* differentiation of DFMSCs into bone-like cells when compared to the CS-only bioaerogel. The nHAp/CS scaffold also showed *in vivo* bone tissue ingrowth over time, leading to higher critical defect fulfillment compared to empty and CS-filled defects, as reported by the longitudinal microCT analysis. Therefore, the bioaerogel showed that it could be an innovative biodegradable bone graft to be applied in low-load-bearing sites such as those found in periodontal and peri-implant bone defects. Future research efforts should focus on further exploring and developing the nHAp/CS scaffold as an alternative material for guiding alveolar bone tissue regeneration *in vivo* in periodontal and peri-implant bone defects since it was able to promote DFMSC osteogenic differentiation.

## Data Availability

The original contributions presented in the study are included in the article/Supplementary Material; further inquiries can be directed to the corresponding author.
